# Association of Antenatal Depression with Adverse Consequences for the Mother and Newborn in Rural Ghana: Findings from the DON Population-Based Cohort Study

**DOI:** 10.1371/journal.pone.0116333

**Published:** 2014-12-30

**Authors:** Benedict Weobong, Augustinus H. A. ten Asbroek, Seyi Soremekun, Alexander A. Manu, Seth Owusu-Agyei, Martin Prince, Betty R. Kirkwood

**Affiliations:** 1 Kintampo Health Research Centre, Ghana Health Service, Kintampo, Ghana; 2 Department of Public Health, Academic Medical Centre, Amsterdam, The Netherlands; 3 Faculty of Epidemiology and Population Health, London School of Hygiene and Tropical Medicine, London, United Kingdom; 4 Health Services and Population Research Department, Institute of Psychiatry, King’s College London, London, United Kingdom; Medical University of Vienna, Austria

## Abstract

**Background:**

Whilst there is compelling evidence of an almost 2-fold increased risk of still births, and suggestive evidence of increased mortality among offspring of mothers with psychotic disorders, only three studies have addressed the role of antenatal depression (AND) on survival of the baby. We examined these associations in a large cohort of pregnant women in Ghana.

**Methods:**

A Cohort study nested within 4-weekly surveillance of all women of reproductive age to identify pregnancies and collect data on births and deaths in the Kintampo Health Research Centre study area of Ghana. Women were screened for AND using the Patient Health Questionnaire (PHQ-9) to ascertain DSM-IV major or minor depression. Outcomes were adverse birth outcomes, maternal/infant morbidity, and uptake of key newborn care practices, examined using logistic regression; effect sizes reported as relative risks with 95% confidence intervals.

**Results:**

20679 (89.6%) pregnant women completed the PHQ-9. The prevalence of AND was 9.9% (n = 2032) (95% confidence interval 9.4%–10.2%). AND was associated with: prolonged labour (RR 1.25, 95% CI 1.02–1.53); peripartum complications (RR 1.11, 95% CI 1.07–1.15);postpartum complications (RR 1.27, 96% CI 1.21–1.34); non-vaginal delivery (RR 1.19, 95% CI 1.02–1.40); newborn illness (RR 1.52, 95% CI 1.16–1.99); and bed net use during pregnancy (RR 0.93, 95% CI 0.89–0.98), but not neonatal deaths, still births, low birth weight, immediate breast feeding initiation, or exclusive breastfeeding. AND was marginally associated with preterm births (RR 1.32, 95% CI 0.98–1.76).

**Conclusion:**

This paper has contributed important evidence on the role of antenatal depression as a potential contributor to maternal and infant morbidity. Non-pharmacological treatments anchored on primary care delivery structures are recommended as an immediate step. We further recommend that trials are designed to assess if treating antenatal depression in conjunction with improving the quality of obstetric care results in improved maternal and newborn outcomes.

## Introduction

Global efforts to reduce the burden of neonatal deaths appear to be yielding positive results with a 1.7 per annum decline rate since 1990 [1]. Nevertheless, sub-Saharan Africa (SSA) has the highest neonatal mortality rate and still accounts for a third of the global deaths as a result of the slow progress in decline [1]. Over 60% of preterm births occur in SSA and South Asia [2], accounting for 27% of all neonatal deaths [3]. Closely related are still births which are similar in numbers to neonatal deaths with 76% occurring in SSA and South Asia [4], but invisible on global policy agendas such as the Millennium Development Goals [5].

Whilst there is compelling evidence of an almost 2-fold increased risk of still births (meta-analysis RR 1.89; 95% CI 1.36–2.62), and suggestive evidence of increased mortality among offspring of mothers with psychotic disorders [6], only three studies in Ethiopia [7], Brazil [8] and the Netherlands [9] have addressed the role of common mental disorders during pregnancy on survival of the baby. All three studies recorded non-statistically significant increased risk of stillbirths or, in the case of the Netherlands, child losses (including stillbirths). The relative risks were; 1.7 (95% CI 0.6–5.5) in Ethiopia, 1.3 (95% CI 0.8–1.9) in the Netherlands, and 1.3 (95% CI 0.4–5.1) in Brazil; all had wide confidence intervals which included 1. Only the study in Ethiopia assessed neonatal mortality and there was no evidence of any increased risk associated with antenatal depression (RR 0.8; 95% CI 0.2–3.0), because the study was underpowered and the ascertainment of neonatal deaths was problematic.

There is, however, strong evidence linking antenatal depression and other adverse birth outcomes such as preterm births (RR 1.13, 95% CI 1.06–1.21) and low birth weight (RR 1.18, 95% CI 1.07–1.30) as reported in a recent meta-analysis involving 26 studies from high income settings and three from low income settings [10]. There was marked heterogeneity of these effect estimates with higher effect sizes for low birth weight (LBW) (RR = 2.06, 95% CI 1.43–2.93) in the two studies from low income countries (Pakistan [11], Brazil [12]). This meta-analysis did not include studies from SSA. Two other studies from low income settings not mentioned in Grote’s meta-analysis reported mixed findings regarding effect sizes for LBW; the study in Bangladesh [13] reported a similar higher effect size as the meta-analysis (OR = 2.24, 95% CI 1.37–3.68), but the study in Brazil [14] found no significant associations with LBW, most likely due to the type of high-risk population studied (pregnant women with a medical condition) which may have masked any effects of antenatal depression. In a more recent review by Davalos and colleagues, of the 8 studies that examined the association between unmedicated depressed mothers at pregnancy and adverse birth outcomes, majority (5 out of 8) reported shorter length of gestation, restricted fetal growth, and/or lower birth weight among neonates of depressed mothers [15]; none of these studies were from SSA, and only 1 was from a low and middle income setting-South Asia. This review did not examine neonatal survival as an outcome because none of the primary studies contributed relevant data. The more recent study in Ethiopia mentioned above found a similar, although non-significant effect, on LBW (RR = 2.3, 95% CI 0.9–6.2), and increased risks for delayed initiation of breastfeeding (more than 8 hours) (RR = 2.8, 95% CI 1.3–6.1) and prolonged labour (more than 24 hours) (RR = 1.6, 95% CI 1.0–2.6) [7]. There is also evidence from mostly high income settings suggesting that antenatal depression is associated with; poor maternal self-care and nutrition, lack of sleep, and inadequate antenatal care [16].

This paper presents findings from a large cohort study conducted in Ghana to address the relative lack of evidence from low and middle income settings, particularly SSA concerning the burden, determinants, and adverse consequences of perinatal depression. The findings presented in this paper include the association of antenatal depression with both adverse birth and maternal morbidity outcomes including birth complications, prolonged labour, and assisted delivery. Finally we present associations with uptake of key newborn care practices.

## Materials and Methods

DON is a cohort study of antenatal and postnatal **D**epression nested within the **O**baapaVitA [17] and **N**ewhints [18] trials in Ghana, conducted within seven contiguous predominantly rural districts in the Brong Ahafo Region. The trials ran consecutively from 2000 to 2009 and collected information on pregnancies, births, and infant and maternal deaths, based on a 4-weekly population-based surveillance system. The ObaapaVitA trial sought to reduce maternal mortality through weekly vitamin-A supplementation of women of reproductive age, and the Newhints trial aimed to assess the impact of home-visits by community health volunteers on neonatal mortality. The area has a population of about 700,000 [19] with more than 120,000 women of reproductive age, and more than 15,000 births a year. There are four large towns (minimum population size of 40,000), with district hospitals. The perinatal mortality rate is 55/1000 live/still births, the neonatal mortality rate is 32/1000 live births, and the stillbirth rate is 31/1000 births [17]. Access to ‘conventional’ mental health services is limited and help for mental ill health is widely provided by traditional healers and spiritual/healing churches [20].

DON was carried out from late January 2008 to early August 2009 and comprised depression assessments in the 4-weekly surveillance visits following identification of pregnancy and in the visits following reporting of a delivery. The analysis in this paper focuses on the consequences of antenatal depression for the mother and baby.

### Data collection

All women of reproductive age were visited at home every 4-weeks by a locally-resident field worker, in order to collect self-reported data on pregnancies, deliveries, including morbidity. When a pregnancy was first reported, information was collected on: socio-demographic and socio-economic indicators, including obstetric history. A DON pregnancy depression assessment was then conducted at the following 4-weekly visit using the Patient Health Questionnaire (PHQ-9). At the first visit after the delivery was reported (usually 4 weeks after delivery), information was collected on the pregnancy, delivery, any complications, the baby (or babies), and new born care practices.

### Exposure

The assessments of antenatal depression were made by administering the Twi (widely spoken language in Ghana and the study area) version of the 9-item Patient Health Questionnaire (PHQ-9) [21]. The PHQ-9 has been previously validated among recently delivered women within the study setting and showed superior psychometric properties when compared with the Edinburgh Postnatal Depression Scale – it recorded a sensitivity of 0.94 and specificity of 0.75 at a cut-off of 5.

The PHQ-9 is a short structured questionnaire that enquires about the nine symptom based criteria for a diagnosis of Diagnostic and Statistical Manual version four (DSM-IV) [22] depression, including duration and severity. This approach allows an approximation to the DSM-IV criteria for major or minor depression, for which only symptoms that have been present for at least half the time in the last two weeks are rated positively. Either depression or anhedonia (loss of interest or pleasure) must be rated, with a total of five or more symptoms for major depression and two to four symptoms for minor depression. In contrast with other symptom based scale scores, these criteria therefore identify individuals with persistent and pervasive symptoms, characteristic of a clinically significant depressive episode. In its initial review it recorded sensitivity and specificity of 0.88 at a cut-off of 10 [22], and high positive predictive value [23].

### Outcomes

We examined three main types of outcomes and these were all reported by the mother at the next 4 weekly visit to the mother after birth. The first is adverse birth outcomes, which covered: neonatal deaths and still births, preterm deliveries (based on the mother’s report on whether the delivery was less than 4 weeks (<37 weeks) before the due date), and any illness that the mother had thought was serious or severe, and low birth weight (LBW) (<2.5 kg). The preterm births (PTB) reported in this study are a result of spontaneous preterm labour. Data collectors were trained to ask this question carefully in order to rule out other possible explanations for the PTB. Birth weight was extracted by fieldworkers from the birth cards given to mothers who delivered in facilities; it was not therefore available for babies delivered at home.

The second type of outcome is maternal morbidity outcomes pertaining to the birth. Women were asked about serious problems they may have experienced during labour/soon after birth or since birth. These were: assisted deliveries (caesarean section and/or instrumental delivery), prolonged labour (23+hrs), peripartum complications (tear in vagina, loss of consciousness, heavy bleeding from vagina, surgery to repair or remove the womb, blood transfusion), and postpartum complications (heavy bleeding/large blood clots from vagina, hot body (high body temperature (>37 C), smelly vaginal discharge, leaking urine/faeces, mastitis, and any other problem not mentioned by the field worker). Data collectors were trained to enquire after these experiences with relevant examples. For example, mastitis is explained as a breast infection that is swollen, painful, or has a discharge, also known as ‘pompo’ in the local language. The list of questions was based on standard maternal morbidity questions which were adapted and piloted in the year 2000 when the surveillance system was established.

The third type is uptake of key newborn care practices: attending at least 4 antenatal care sessions, initiating breast feeding within an hour of birth, exclusively breast feeding within the first month based on the mother’s account of breast feeding in the last 24 hours.

#### Potential confounders

A priori potential confounders were: maternal characteristics (age, marital status, education status, occupation, ethnicity, maternal malaria, religion and rural or urban residence); pregnancy and obstetric variables (parity). In addition, an overall socio-economic ‘score’ for each woman was generated using factor analysis techniques after the methods described by Vyas and Kumaranayake (2006) [24], and the detailed account of this is reported in our companion paper on determinants of postnatal depression [25]. Briefly individual asset factor scores were summed for each woman to provide a measure of her overall socio-economic score, where the higher the score, the higher the assumed economic status of the household. Women were ranked according to these socio-economic scores into wealth quintile groups. We also took into consideration the potential confounding effect of the two trials on the outcomes of this study.

### Power considerations

Over 20,000 pregnant women were screened for depression. With the prevailing rates of neonatal mortality and still births within the study population, coupled with the sample sizes available and antenatal depression prevalence, we estimated to be able to detect an effect size of 1.23 with 95% confidence intervals and 80% power.

### Statistical analyses

Analyses were based on women who had a DON depression screening during pregnancy, and their babies, and were restricted to singletons. Multiple pregnancies were excluded from all analyses given the high risk associated with infant mortality particularly in Africa [26]. The association between antenatal depression and each of the outcomes was carried out using logistic regression models adjusting for a priori confounders, including intervention effect, given this cohort was nested within two consecutive trials. Effect sizes are reported as relative risks, with 95% confidence intervals and p-values using the marginal standardization technique to estimate these from odds ratios via the delta method [27]. All analysis were conducted using STATA 11 [28].

### Ethical considerations

Ethical approval for the study was granted by the ethics committees of the Kintampo Health Research Centre (KHRC) and the London School of Hygiene and Tropical Medicine. Written informed consent was obtained from all participants using an informed consent form that was approved by the ethics committees. The informed consent procedure was either conducted in English or the local language (Twi) for those women who were not literate in English; in such instances a literate witness was involved.

## Results

**Figure 1:**
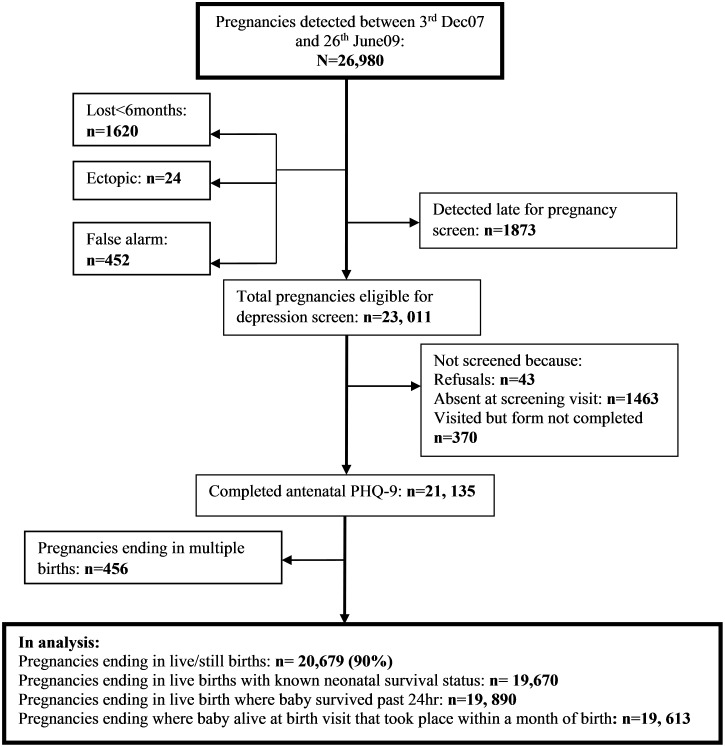
Recruitment profile. 1. **Lost<6 months:** pregnancies lost before 6 months gestation. **2. Ectopic:** tubal pregnancy. **3. False alarm:** false report of a pregnancy by the mother. **4. PHQ-9:** Patient Health Questionnaire.

**Fig. 1** shows the recruitment profile. Between 3^rd^ December 2007 and 25^th^ June 2009, 26,980 pregnant women were identified, of whom 23,011 were eligible for depression assessment. Of these 20,679 (89.8%) pregnant women with a singleton birth (live/still) completed the depression screen. This is the denominator used for analyses examining maternal morbidity outcomes and pregnancy behaviours, and still births. Forty-three (0.2%) declined to participate, 1463 (6.4%) were temporarily absent at the surveillance visit, and 370 (1.6%) did not have a depression form completed although they were visited. Background characteristics of those not met and screened were comparable to those in the analysis (see companion paper [29]). Three other denominators are also shown: the number of live births with known neonatal survival status (19,670) used for determination of neonatal mortality, the number surviving past the first 24 hours (19,890) used for estimation of initiation of breast feeding, and the number of babies still alive within a month after birth (19,613) used for exclusive breast feeding, bed net use, and severe newborn illness outcomes.

The prevalence of DSM-IV major or minor depression during pregnancy was 9.9% (n = 2032) (95% confidence interval 9.4%–10.2%). Detailed profile of study participants is given in a companion paper [29]. In brief the population was predominantly rural (70%) and the modal age group was 20–29 (53%). Almost all the women were married (91%), most had some education (64%), and most belonged to the Christian faith (68%).

### Risk of Adverse Perinatal and Neonatal Outcomes

**Table 1** shows that only severe newborn illness (Adjusted RR 1.52, 95% CI 1.16–1.99) was significantly increased among mothers with antenatal depression. The evidence for the association between antenatal depression and risk of preterm delivery (Adjusted RR 1.32, 95% CI 0.98–1.76) was weak. There was no evidence of associations with neonatal mortality, still birth, or LBW.

**Table 1:** Effect of antenatal depression on risk of adverse perinatal/neonatal outcomes.

Outcome	Numberof babies	n (% withoutcome)	Crude Relativerisk (95% CI)	Adjusted Relativerisk (95% CI)	p-value
† **Neonatal mortality**					
Not Depressed group	17424	421 (2.4%)	1	1	
Depressed group	1883	47 (2.5%)	1.03 (0.77–1.39)	1.02 (0.76–1.37)	0.918
# **Still births**					
Not Depressed group	18358	453 (2.5%)	1	1	
Depressed group	1995	54 (2.7%)	1.10 (0.83–1.45)	1.06 (0.80–1.40)	0.673
## **Preterm births**					
Not Depressed group	15290	369 (2.4%)	1	1	
Depressed group	1590	50 (3.1%)	1.30 (0.97–1.74)	1.32 (0.98–1.76)	0.065
* **Low birth weight**					
Not Depressed group	9917	702 (7.1%)	1	1	
Depressed group	1031	65 (6.3%)	0.89 (0.70–1.14)	0.87 (0.69–1.11)	0.262
## **Severe Newborn illness**					
Not Depressed group	17479	369 (2.1%)	1	1	
Depressed group	1890	60 (3.2%)	1.50 (1.15–1.96)	1.52 (1.16–1.99)	0.002

†Expressed ‰ live births, restricted to babies with survival status at end of neonatal period known (424 neonates were lost to 28 day follow up). Adjusted for: woman’s age, education, wealth quintile, marital status, area of residence, ethnicity, religion, parity, perceived birth weight, baby’s sex, initiation of breastfeeding, delivery place, and intervention effect.

#Expressed ‰ live+still births. Adjusted for: woman’s age, education, wealth quintile, marital status, area of residence, ethnicity, religion, parity, previous still birth, baby’s sex, malaria, and intervention effect.

*LBW<2.5 kg: data available only for hospital deliveries. Adjusted for: woman’s age, education, wealth quintile, marital status, area of residence, ethnicity, religion, parity, preterm birth, malaria, and intervention effect.

##PTB<37 weeks gestation: Adjusted for: woman’s age, education, wealth quintile, marital status, area of residence, ethnicity, religion, parity, baby’s sex, malaria, and intervention effect.

### Risk of Adverse Maternal Outcomes (Morbidity)

**Table 2** shows the risk of maternal morbidity associated with antenatal depression. Risk of severe peripartum complications (Adjusted RR 1.11, 95% CI 1.07–1.15 p<0.001); postpartum complications (Adjusted RR 1.27, 95% CI 1.21–1.34 p<0.001); caesarean section and/or instrumental delivery (Adjusted RR 1.19, 95% CI 1.02–1.40 p = 0.032); and prolonged duration of labour (Adjusted RR 1.25, 95% CI 1.02–1.53 p = 0.028), were all significantly elevated among mothers with antenatal depression. Further analysis showed that four of the eight peripartum complications were more likely to be reported by depressed women antenatally (**S1 Table**); these were heavy bleeding (Adjusted RR 1.27, 95% CI 1.18–1.38 p<0.001), tear in the vagina (Adjusted RR 1.19, 95% CI 1.08–1.30 p<0.001), placenta replacement (Adjusted RR 1.17, 95% CI 1.06–1.29 p = 0.002), and convulsions (Adjusted RR 1.74, 95% CI 1.04–2.93 p = 0.036). Further analysis also showed that postpartum complications reported were significantly elevated among antenatally depressed women, with the biggest effects on hot body (Adjusted RR 1.52, 95% CI 1.34–1.72 p<0.001), other serious complications (Adjusted RR 1.49, 95% CI 1.29–1.73 p<0.001), and leaking urine/faeces (Adjusted RR 1.39, 95% CI 1.14–1.70 p = 0.001) (**S2 Table**).

**Table 2:** Effect of antenatal depression on poor birth outcomes including morbidity among mothers with singleton births.

Outcome	Number of women witha singleton birth anddepression record (n)	n (% with outcome)	Crude Relative risk (95% CI)	Adjusted Relative risk (95% CI)	p-value
‡ **Peripartum complications**					
Not Depressed group	18095	9901 (54.7%)	1	1	
Depressed group	1962	1184 (60.4%)	1.10 (1.06–1.15)	1.11 (1.07–1.15)	<0.001
‡ **Postpartum complications**					
Not Depressed group	18198	6516 (35.8%)	1	1	
Depressed group	1970	917 (46.6%)	1.30 (1.24–1.37)	1.27 (1.21–1.34)	<0.001
† * **Prolonged labour (24+ hours)**					
Not Depressed group	5994	650 (10.8%)	1	1	
Depressed group	696	94 (13.5%)	1.25 (1.02–1.52)	1.25 (1.02–1.53)	0.028
* **CS and/or Instrumental delivery**					
Not Depressed group	18462	1254 (6.8%)	1	1	
Depressed group	2006	152 (7.6%)	1.12 (0.95–1.31)	1.19 (1.02–1.40)	0.032

‡ Adjusted for: woman’s age, education, wealth quintile, marital status, area of residence, ethnicity, religion, parity, previous mode of delivery, delivery place, preterm birth, and intervention effect.

*Adjusted for: woman’s age, education, wealth quintile, marital status, area of residence, ethnicity, religion, parity, and intervention effect.

† data available only on a sub-sample of the cohort studied.

### Risk of Pregnancy Behaviours and Newborn Care Practices

**Table 3** shows that women with antenatal depression were significantly less likely to have reported using a bed net during pregnancy (Adjusted RR 0.93, 95% CI 0.89–0.98 p = 0.005). There was however no evidence that they were less likely to put their neonate under a bed net (Adjusted RR 1.01, 95% CI 0.98–1.04 p = 0.479). There was also no evidence of association between antenatal depression and antenatal care attendance, delivering at a health facility, immediate initiation of breastfeeding, or exclusive breastfeeding within the neonatal period.

**Table 3:** Effect of antenatal depression on the uptake of selected key newborn care practices.

Outcome	Number withdepression record (n)	n (% with outcome)	Crude Relative risk (95% CI)	Adjusted Relative risk (95% CI)	p-value
## **Antenatal care attendance (>4 times)**					
Not Depressed group	18115	12973 (71.6%)	1	1	
Depressed group	1965	1374 (69.9%)	0.98 (0.95–1.01)	1.01 (0.98–1.03)	0.625
## **Bed net use during pregnancy**					
Not Depressed group	18452	9174 (49.7%)	1	1	
Depressed group	2003	929 (46.4%)	0.93 (0.89–0.98)	0.93 (0.89–0.98)	0.005
## **Delivering at health facility**					
Not Depressed group	18462	12465 (67.5%)	1	1	
Depressed group	2006	1293 (64.5%)	0.95 (0.92–0.99)	1.00 (0.97–1.03)	0.905
## * **Bed net use for baby**					
Not Depressed group	12918	9797 (75.8%)	1	1	
Depressed group	1425	1079 (75.7%)	1.00 (0.97–1.03)	1.01 (0.98–1.04)	0.479
# ** **Initiation of breast feeding (<1 hour)**					
Not Depressed group	13098	6109 (46.4%)	1	1	
Depressed group	1443	654 (45.3%)	0.97 (0.92–1.03)	1.00 (0.95–1.06)	0.897
## * **Exclusive Breast Feeding**					
Not Depressed group	12918	11255 (87.1%)	1	1	
Depressed group	1425	1233 (86.5%)	0.99 (0.97–1.02)	0.99 (0.97–1.02)	0.609

#adjusted for: woman’s age, education, wealth quintile, marital status, area of residence, ethnicity, religion, parity, place of delivery, mode of delivery, and intervention effect.

##Adjusted for: woman’s age, education, wealth quintile, marital status, area of residence, ethnicity, religion, parity, and intervention effect.

*Number of singleton live babies within 4 weeks of delivery.

**All singleton live births up to 24 h after birth, and visited within 4 weeks of delivery.

## Discussion

This is the largest cohort study that has yet been conducted in low income settings of the effects of antenatal depression on adverse outcomes for the mother and baby. After adjustment for confounders, antenatal depression was not found to be associated with neonatal deaths, still births, low birth weight, delayed initiation of breastfeeding, or non-exclusive breastfeeding in the neonatal period, delivering at a health facility, or optimal antenatal care attendance. However, antenatal depression was associated with a 25% increase in 24+ hours prolonged labour, 11% severe peripartum and 27% postpartum complications, 50% severe newborn illness, and 7% less bed net non-use during pregnancy. It was also marginally associated with a 32% increase risk of preterm deliveries.

### Strengths and weaknesses of the study

Our report is strengthened by several factors. We employed an unprecedentedly large cohort of 20,679 pregnant women, applied clinimetric criterion for depression using a locally validated tool, ascertained outcomes blind to exposures because the data collectors were not aware of the study hypothesis, and recorded a high antenatal depression screening response rate of 92%. Further to this, we employed a more robust ascertainment of neonatal deaths based on the frequent 4-weekly surveillance visits to mothers. Our measure of low birth weight is also robust as these were recorded at the health facility.

Possible weaknesses are our self-reported morbidity outcomes may have been influenced by the mother’s depression status after birth; however this may not have had the assumed biased effect as 87% of women depressed antenatally will usually not be depressed at their postpartum assessment in this setting [25]. Furthermore, although we accounted for confounding in our analyses, we did not have data on maternal Body Mass Index and intimate partner violence during pregnancy both of which are known to be associated with both adverse perinatal outcomes [30], [31] and antenatal depression [7], [8], [32], [33]. Maternal smoking is also a well-established risk factor for both antenatal depression [34] and adverse birth outcomes [35], but we were unable to measure the effect of this confounder in our analysis because of lack of data. We argue that smoking in women is culturally frowned upon in the study setting and thus would have been challenging to elicit through the questionnaire method. In addition, smoking behaviour is relatively low in this setting and the effect of second hand smoke exposure can be argued to be minimal.

In addition our measure of antenatal depression was not originally validated in the antenatal period and given that one study suggests depression in pregnancy and postnatal period show significantly different symptom profiles [36], the prevalence of the exposure may have been underestimated.

### Comparison with other studies

Our finding of an independent association between poor antenatal mental health and prolonged labour is consistent with findings from both LMIC [7] and high income settings [37], [38]. There are reasons why antenatal depression may be associated with prolonged labour and other obstetric complications. First there is evidence suggestive of a direct effect of psychoneuroendocrine processes upon obstetric complications and foetal outcomes resulting from antenatal psychosocial stress [39]–[41]. Secondly, worry about the outcome of pregnancy is understandably common in settings with a high burden of maternal mortality and other adverse birth outcomes. The association between antenatal depression and new-born illness demonstrated in our study is suggestive of suboptimal care provided by the depressed mother in the early neonatal period as similarly highlighted in a study in Ethiopia [7] and is consistent with findings from other studies that explored this relationship with older infants in South Asia [42], [43]. In our study, the fact that antenatal depression resulted in 7% of mothers being unable to use the insecticide treated bed net highlights another finding of public health significance particularly in SSA and other regions where malaria is endemic. The insecticide treated bed net is a WHO intervention aimed at preventing malaria in areas with high populations of the main malaria vector (infected mosquito), and pregnant mothers are strongly advised to sleep under the insecticide treated net in order to avoid contracting malaria and thus ensure the optimal development of the foetus.

The evidence on the association between antenatal depression and poor birth outcomes including the survival of the baby in low and middle income settings is scanty. We are aware of only one study in SSA (Ethiopia) that examined the association between antenatal depression and neonatal mortality, and this showed no evidence of any increased risk [7], similar to our finding. We also found no evidence for an increased risk of still births among antenatally depressed women, and our point estimate of 1.06 was lower than those reported in Ethiopia (1.7) [7], Brazil (1.3) [8], and the Netherlands (1.3) [9] all of which also had wide confidence intervals including one. Our non-significant increased risk of preterm births (1.32) is higher than the meta-analysis estimate of 1.13 from low income settings [10].

Our negative finding on LBW, is in contrast to the increased risks found in other cohort studies in developing countries in Ethiopia [7], India [44], Pakistan [45], and Brazil [8]. One difference between these studies and ours is that our estimate is restricted to women delivering in facilities; however selection bias is unlikely to explain the difference because a high proportion (67%) delivered in the facilities and this was the same for depressed and non-depressed women.

Our study did not also replicate the positive association between antenatal common mental disorder and delayed initiation of breastfeeding in Ethiopia (2.8, 95% CI 1.3–6.1) [7]. Our findings may be due to the fact that breastfeeding uptake within the immediate postnatal period is generally high in our setting (86% were exclusively breast feeding within a month of delivery).

Though perinatal depression has been shown to affect the health-related quality of life of the mother [46], there are few accounts of its association with specific serious medical complications during labour or soon after delivery. Our finding of an association with both severe peripartum and postpartum complications suggests that poor maternal mental health may have a role as a potential contributor to maternal deaths in regions of high burden, but this hypothesis requires further investigation.

### Implications

Although antenatal depression in our setting and SSA in general may be self-limiting [25], [47], [48], it may have serious consequences for both the mother and baby, and interventions are encouraged. Bearing in mind however the potential risks of pharmacological treatment in pregnancy [49]–[51], we would recommend psychosocial/psychological treatment options as the first step for women with antenatal depression as prescribed in the mental health gap action programme guidelines (mhGAP-IG) [52]. Specific findings reported in this paper also suggest that efforts are put in place to systematically identify mothers who are depressed during pregnancy, and to use this knowledge to inform closer antenatal and delivery care whereby mothers are encouraged to deliver at health facilities equipped to deal with obstetric complications. Further, given recent evidence suggesting that therapies that target the mother-infant relationship are efficacious in tackling detrimental consequences for children of depressed mothers [53], studies/trials are urgently required to examine whether treating antenatal depression in conjunction with improving the quality of obstetric care would lead to improved birth outcomes.

### What is already known about this topic?

Antenatal depression is associated with:

- Low birth weight/stunted growth particularly in south Asia
- Preterm births
- Prolonged labour
- Postnatal depression
- Poor maternal self-care and nutrition

### What this study adds

Antenatal depression is associated with:

- Severe newborn illness
- Bed net non-use during pregnancy
- Severe peripartum and postpartum complications
- Instrumental/caesarean section

## Supporting Information

S1 Table **Effect of antenatal depression on risk of specific peripartum complications.**
(DOCX)

S2 Table **Effect of antenatal depression on risk of specific postpartum complications.**
(DOCX)
