# Supplementary material for: Association of Antenatal Depression with Adverse Consequences for the Mother and Newborn in Rural Ghana: Findings from the DON Population-Based Cohort Study
Source: PLoS One. 2014 Dec 30;9(12):e116333. doi: 10.1371/journal.pone.0116333 (PMC4280205; doi:10.1371/journal.pone.0116333)
Supplement: S1 Table — Effect of antenatal depression on risk of specific peripartum complications. (DOCX) [file pone.0116333.s001.docx]

| **Outcome** | **Number with depression record (n)** | **n (% with outcome)** | **Crude Relative risk (95%CI)** | ***Adjusted Relative risk (95%CI)** | **p-value** |
| --- | --- | --- | --- | --- | --- |
| **Heavy bleeding** | | | | | |
| Not Depressed group | 18203 | 3866 (21.2%) | 1 | 1 |  |
| Depressed group | 1972 | 554 (28.1%) | 1.32 (1.23-1.43) | 1.27 (1.18-1.38) | <0.001 |
| **Tear in vagina** | | | | | |
| Not Depressed group | 18200 | 3043 (16.7%) | 1 | 1 |  |
| Depressed group | 1975 | 378 (19.1%) | 1.14 (1.04-1.26) | 1.19 (1.08-1.30) | <0.001 |
| **Placenta replacement** | | | | | |
| Not Depressed group | 17978 | 2823 (15.7%) | 1 | 1 |  |
| Depressed group | 1943 | 354 (18.2%) | 1.16 (1.05-1.28) | 1.17 (1.06-1.29) | 0.002 |
| **Convulsions** | | | | | |
| Not Depressed group | 18206 | 86 (0.5%) | 1 | 1 |  |
| Depressed group | 1973 | 17 (0.9%) | 1.82 (1.09-3.06) | 1.74 (1.04-2.93) | 0.036 |
| **Loss of consciousness** | | | | | |
| Not Depressed group | 18012 | 271 (1.5%) | 1 | 1 |  |
| Depressed group | 1945 | 41 (2.1%) | 1.31 (0.94-1.82) | 1.36 (0.99-1.89) | 0.062 |
| **IV drip** | | | | | |
| Not Depressed group | 18224 | 4633 (25.4%) | 1 | 1 |  |
| Depressed group | 1975 | 499 (25.3%) | 0.99 (0.92-1.08) | 1.03 (0.96-1.11) | 0.362 |
| **Surgery** | | | | | |
| Not Depressed group | 18152 | 711 (3.9%) | 1 | 1 |  |
| Depressed group | 1968 | 76 (3.8%) | 0.99 (0.78-1.24) | 0.99 (0.79-1.24) | 0.910 |
| **Blood transfusion** | | | | | |
| Not Depressed group | 18219 | 377 (2.1%) | 1 | 1 |  |
| Depressed group | 1975 | 41 (2.1%) | 1.00 (1.73-1.38) | 0.99 (0.72-1.35) | 0.926 |

*adjusted for: woman’s age, education, wealth quintile, marital status, area of residence, ethnicity, religion, parity, previous mode of delivery, delivery place, perceived size of baby, preterm birth, and intervention effect.
