# Supplementary material for: Association of Antenatal Depression with Adverse Consequences for the Mother and Newborn in Rural Ghana: Findings from the DON Population-Based Cohort Study
Source: PLoS One. 2014 Dec 30;9(12):e116333. doi: 10.1371/journal.pone.0116333 (PMC4280205; doi:10.1371/journal.pone.0116333)
Supplement: S2 Table — Effect of antenatal depression on risk of specific postpartum complications. (DOCX) [file pone.0116333.s002.docx]

| **Outcome** | **Number with depression record (n)** | **n (% with outcome)** | **Crude Relative risk (95%CI)** | ***Adjusted Relative risk (95%CI)** | **p-value** |
| --- | --- | --- | --- | --- | --- |
| **Hot body** | | | | | |
| Not Depressed group | 18219 | 1522 (8.4%) | 1 | 1 |  |
| Depressed group | 1975 | 261 (13.2%) | 1.58 (1.40-1.79) | 1.52 (1.34-1.72) | <0.001 |
| **Other problems** | | | | | |
| Not Depressed group | 18224 | 1114 (6.1%) | 1 | 1 |  |
| Depressed group | 1975 | 189 (9.6%) | 1.57 (1.35-1.81) | 1.49 (1.29-1.73) | <0.001 |
| **Leaking urine/faeces** | | | | | |
| Not Depressed group | 18214 | 707 (3.9%) | 1 | 1 |  |
| Depressed group | 1971 | 104 (5.3%) | 1.36 (1.11-1.66) | 1.39 (1.14-1.70) | 0.001 |
| **Mastitis** | | | | | |
| Not Depressed group | 18223 | 1106 (6.1%) | 1 | 1 |  |
| Depressed group | 1975 | 170 (8.6%) | 1.42 (1.22-1.66) | 1.38 (1.18-1.61) | <0.001 |
| **Large clots/heavy bleeding** | | | | | |
| Not Depressed group | 18203 | 3444 (18.9%) | 1 | 1 |  |
| Depressed group | 1973 | 505 (25.6%) | 1.35 (1.25-1.47) | 1.32 (1.21-1.43) | <0.001 |
| **Vaginal discharge** | | | | | |
| Not Depressed group | 18196 | 1090 (5.9%) | 1 | 1 |  |
| Depressed group | 1971 | 156 (7.9%) | 1.32 (1.12-1.55) | 1.27 (1.08-1.50) | 0.003 |

*adjusted for: woman’s age, education, wealth quintile, marital status, area of residence, ethnicity, religion, parity, previous mode of delivery, place of delivery, preterm birth, and intervention effect.
